# Metabolomic analysis of vascular cognitive impairment due to hepatocellular carcinoma

**DOI:** 10.3389/fneur.2022.1109019

**Published:** 2023-03-16

**Authors:** Dan Zhu, Yamei Zhu, Lin Liu, Xiaoxue He, Shizhong Fu

**Affiliations:** ^1^Xinqiao Hospital, Army Medical University (Third Military Medical University), Chongqing, China; ^2^Deptartment of Infectious Diseases, Wuhua Ward, 920th Hospital of Joint Logistics Support Force of Chinese PLA, Kunming, Yunnan, China; ^3^Dalian Hunter Information Consulting Co. LTD, Dalian, China

**Keywords:** metabolomics, metabolic DEGs, prognostic model, biomarkers, neurovascular disease, hepatocellular carcinoma (HCC), vascular cognitive impairment (VCI)

## Abstract

**Introduction:**

Screening for metabolically relevant differentially expressed genes (DEGs) shared by hepatocellular carcinoma (HCC) and vascular cognitive impairment (VCI) to explore the possible mechanisms of HCC-induced VCI.

**Methods:**

Based on metabolomic and gene expression data for HCC and VCI, 14 genes were identified as being associated with changes in HCC metabolites, and 71 genes were associated with changes in VCI metabolites. Multi-omics analysis was used to screen 360 DEGs associated with HCC metabolism and 63 DEGs associated with VCI metabolism.

**Results:**

According to the Cancer Genome Atlas (TCGA) database, 882 HCC-associated DEGs were identified and 343 VCI-associated DEGs were identified. Eight genes were found at the intersection of these two gene sets: NNMT, PHGDH, NR1I2, CYP2J2, PON1, APOC2, CCL2, and SOCS3. The HCC metabolomics prognostic model was constructed and proved to have a good prognostic effect. The HCC metabolomics prognostic model was constructed and proved to have a good prognostic effect. Following principal component analyses (PCA), functional enrichment analyses, immune function analyses, and TMB analyses, these eight DEGs were identified as possibly affecting HCC-induced VCI and the immune microenvironment. As well as gene expression and gene set enrichment analyses (GSEA), a potential drug screen was conducted to investigate the possible mechanisms involved in HCC-induced VCI. The drug screening revealed the potential clinical efficacy of A-443654, A-770041, AP-24534, BI-2536, BMS- 509744, CGP-60474, and CGP-082996.

**Conclusion:**

HCC-associated metabolic DEGs may influence the development of VCI in HCC patients.

## Introduction

Hepatocellular carcinoma (HCC) is one of the most common malignant tumors worldwide, characterized by the insidious and rapid onset, poor prognosis, and high metastasis rate. With an estimated 906,000 new cases of HCC in 2020, accounting for 4.7% of the overall cancer incidence, HCC is the sixth most common malignancy worldwide, after breast, lung, colorectal, prostate, and stomach cancers (1). It is the third most common cause of cancer-related death and patients generally have a poor prognosis as the onset is insidious and the tumor is often at an advanced stage at the time of diagnosis. In 2020, ~830,000 people died from HCC globally, accounting for 8.3% of cancer-related deaths (1). In recent years, with the popularization of hepatitis B vaccination and the improved efficacy of antiviral drug therapy, the incidence of virus-related HCC is on the decline, while non-alcoholic liver disease and metabolic syndrome-related HCC are increasing year by year due to lifestyle changes and dietary habits (2). The main treatment options for HCC include surgical resection, radiofrequency ablation, chemotherapy, liver-targeted drugs, and liver transplantation. However, due to the insidious onset of the disease, the early stages may present with only non-specific symptoms such as weakness and dyspepsia, and progresses rapidly, resulting in the diagnosis of the tumor at an advanced stage, by when the opportunity for radical treatment had already passed (3). The late stage of HCC is often associated with complications such as gastrointestinal bleeding, infection, hepatic and renal syndrome, and hepatic encephalopathy. When complicated by hepatic encephalopathy, clinical manifestations may include behavioral abnormalities, cognitive impairment, altered consciousness, and even coma, which are often combined with liver dysfunction making the treatment more difficult and the patient's prognosis poor (4, 5). However, no extensive study has been conducted to determine whether other factors may contribute to cognitive impairment in patients with HCC.

Vascular cognitive impairment (VCI) is a syndrome of cognitive impairment secondary to bleeding, ischemic stroke, cerebrovascular injury, and other diseases. Patients may present with cognitive impairment such as memory problems, behavioral abnormalities, speech difficulties, and even dementia (6). Various mechanisms involving immune inflammatory response, oxidative stress, and damage to the blood-brain barrier are associated with the development of VCI (7–11). Some studies have also reported that a strong correlation between HCC and the changes in intestinal microbiota in Alzheimer's disease has been observed, indicating that HCC may promote cognitive impairment in Alzheimer's disease by affecting the intestinal microbial ecology (12). Furthermore, some studies have found common transcriptional changes between Alzheimer's disease and HCC and other cancers (13). Abnormal levels of circulating metabolites such as amino acids and fatty acids are associated with cognitive impairment caused by vascular dysfunction (14–16). Metabolomic analysis showed that dysregulation of various metabolites was closely related to HCC, suggesting its involvement in promoting the occurrence of HCC-related VCI (17). The circulating metabolites may, therefore, be affected by HCC, thereby resulting in cognitive impairment.

Metabolomics is the qualitative and quantitative analysis of the metabolites of an organism or cell during a specific period to deduce the relationship between different metabolites and the corresponding pathophysiological state. Metabolomics is now widely used in many fields such as disease diagnosis, drug toxicology, pharmaceutical development, and microbial metabolism (18, 19). Since abnormal metabolism is a common feature of cancer cells, metabolomics plays an important role in tumor prognosis, drug target research, and metabolic marker screening (20). The liver is the central organ of human metabolism and is involved in regulating the expression levels of many metabolites, which makes the metabolomic study of HCC particularly important (21, 22). Zoe Hall and other researchers found that the proliferation of HCC tumor cells is closely related to altered metabolic pathways such as adipogenesis and phosphatidylcholine production (23). Further, the serum bile acid levels were significantly higher and serum sphingolipid levels were lower in HCC patients, suggesting that the changes in these metabolites are closely related to the development of HCC (24). Metabolomic analysis of VCI showed that the disease-related biomarkers were mainly associated with homocysteine, folate, branched-chain amino acids, and lipid metabolism (16). Sphingolipids, cholesterol, phospholipids, and other lipids play an important role in the maintenance of the structure and function of neuronal structures. Therefore, some researchers have suggested that disorders of lipid metabolism could be vital in the pathogenesis of VCI, but the exact mechanism is not yet clear (25). This suggests that HCC and VCI are associated with metabolomic changes, and related studies are needed.

The development of next generation sequencing (NGS) technologies and bioinformatic tools allows a large-scale analysis of each parameter involved in cancer and other systemic disease (26–32). In this study, the Gene Expression Omnibus (GEO) database and literature search were utilized to obtain metabolomic data on HCC and VCI, and Metaboanalyst 5.0 was used to obtain the metabolic differentially expressed genes (DEGs) as previous researches (33–36). In order to understand the possible mechanism of VCI caused by HCC and provide some theoretical basis for the clinical treatment of HCC, we conducted principal component analyses, functional enrichment analyses, immune function analyses, and tumor mutational burden (TMB) analyses of the above genes. We investigated possible mechanisms of VCI induction by HCC in this study.

## Methods

### Data acquisition

VCI clinical and transcriptomic (mRNA) data were downloaded from GEO database. The filter conditions were set to ①” vascular cognitive impairment”; ② human. This study was derived from 10 “normal cognitive patients” and 10 “patients with VCI” from the microarray dataset GSE201482 in 10 cases. DEGs of VCI were screened according to the criteria of *P*-value < 0.05 and |log_2_FC| ≥ 1.00. HCC clinical, transcriptomic (mRNA) data were downloaded from TCGA (https://portal.gdc.cancer.gov/) website, and their expression matrix was compiled and summarized by R. DEGs were screened by adjust *P*-value < 0.05 and |log_2_FC| ≥ 1.0, and heat maps were plotted. DEGs were categorized into up-regulated, down-regulated, and non-statistically significant groups, and then imported into R for volcano plots. Bioconductor R software's bioconductor R package was used to normalize and calculate expression values for microarray data. Based on the screened DEGs, heat maps and cluster analyses were performed using the heatmap package. We transformed DEG *P*-value to –log10, grouped them according to log_2_FC, and imported the processed data into R for volcano plotting. The HCC expression matrix data was downloaded from the Cancer Genome Atlas database. According to the pre-screened genes, the relative expression of the core genes in the HCC expression data was analyzed using “ggpubr” package and a relative expression box plot was drawn.

### Metabolomics matrix construction and multi-omics analyses

For multi-omics analysis, metabolically differentially expressed metabolites and DEGs were imported from HCC and VCI metabolomics into Metaboanalyst 5.0, while metabolomics genes associated with HCC and VCI were incorporated into Venny 2.1 software (http://bioinfogp.cnb.csic.es/tools/venny/index.html) for plotting Venn diagrams and obtaining metabolomic differential genes associated with HCC and VCI.

### Immuno-infiltration analysis and immunofunctional analysis

The HCC expression matrix data obtained above were subjected to a deconvolution algorithm using the CIBERSORT package, which allows estimation of the cellular composition of complex tissues based on normalized gene expression data, as well as quantification of specific cell types. The immune cell sorting perl script is used to sort the HCC-associated infiltrating immune cells. The limma R package in R was used to calculate expression values from the microarray data. HCC and paraneoplastic tissues were analyzed using the CIBERSORT package. With the CIBERSORT package, the composition of immune cells in each sample was further analyzed and histograms were plotted. Pheatmap was used to generate heat maps showing immune cell distribution. HCC immune cell infiltration co-expression map was plotted using corrplot to analyse interactions between immune cell populations. Finally, the expression of each immune cell was analyzed using the vioplot package in HCC tissues and paracancerous tissues. In relation to immunofunctional analysis, we used the “limma,” “GSVA,” “gseabase,” “pheatmap,” and “reshape2” packages to perform immunofunctional analysis on HCC metabolomics-related genes to identify targets for precision therapy.

### VCI-associated HCC metabolic genes prognostic model

Standardized HCC metabolomics data were merged with HCC clinical data, and univariate and multifactorial Cox prognostic survival analyses on HCC differential genes were performed using the R language packages survival, caret, glmnet, survminer, and survroc to plot survival curves for closely related key metabolomics genes. In addition, 370 cases were randomly divided into testing group (*n* = 185) and training group (*n* = 185). R software was used to perform risk survival analysis on the pre-merged data, plotting risk survival curves and receiver operator characteristic (ROC) curves for testing group, training group, and total group as previous studies (37–40). On the general clinical data of HCC, the survival package was again used to perform clinical statistical analysis and risk prognostic analysis. To investigate the risk factors associated with HCC, forest plots, and histograms were plotted for single- and multi-factor independent prognostic analysis. Further model validation on clinical subgroups was performed using the “survival” and “survminer” packages.

### Principal component analysis and enrichment analysis of gene ontology (GO) and the Kyoto encyclopedia of genes and genomes (KEGG) for genes associated with HCC metabolomics

The scatterplot3d package was used for the principal component analysis of HCC metabolomics-related genes and HCC-related risk genes, and the clusterprofilergo.R package in R (https://www.r-project.org/) software and Perl language were used for metabolomics-related HCC GO analysis for differential genes. KEGG pathway enrichment analysis was performed using the clusterprofilerkegg.R package to analyze core pathway enrichment based on enrichment factor values and to investigate the biological functions and signaling pathways that may be associated with HCC.

### TMB analysis of HCC metabolism-related genes

TMB files were downloaded from the TCGA database and correlation tests between core genes and TMB were performed using functions, with correlation coefficients and *P*-values calculated. In addition, correlation analysis between metabolism-related genes and HCC tumor mutational load was performed using the survival and survminer software packages, and correlation coefficients and *P*-values were calculated.

### Gene set enrichment analysis (GSEA)

The GSEA website was used to download the GO/KEGG annotation files for the whole transcriptome genes, and GO and KEGG enrichment analyses of the core genes were performed using the limma, org. Hs. eg. db, clusterprofiler, and enrichplot packages as previous researches (41–43). Analyses of cellular component (CC), molecular function (MF), biological process (BP), and KEGG pathway enrichment were conducted for the core genes. Based on the enrichment factor values, we analyzed the core pathway enrichment and examined the potential biological functions and signaling pathways of the HCC core genes.

### Screening for potential therapeutic drugs

“pRRophetic” is a package that can be used to predict phenotypes from gene expression data, predict drug sensitivity in external cell lines, and predict clinical data. In order to determine the drug sensitivity of each sample from TCGA database, we used the R package “pRRophetic” to obtain HCC metabolism-related genes after prescreening.

## Results

### Metabolites with differential levels in patients with HCC and VCI

**Figure 1:**
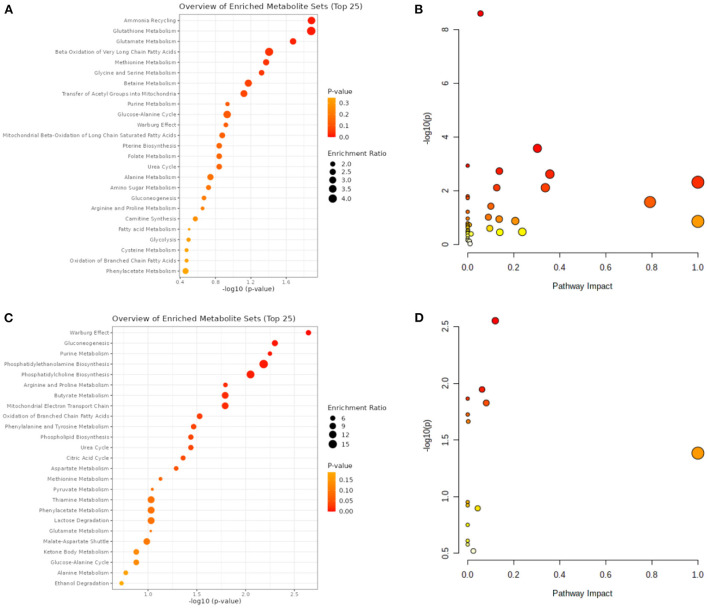
VCI and HCC metabolomic analysis. **(A)** VCI differentially expressed metabolite enrichment analysis diagram; **(B)** Differentially expressed metabolite signaling pathways in VCI; **(C)** Differentially expressed metabolite enrichment distributions in HCC; **(D)** Differentially expressed metabolite signaling pathways in HCC.

A total of 14 differentially expressed metabolites from HCC, including 2 up-regulated and 12 down-regulated metabolites, as well as 71 differentially expressed metabolites from VCI, including 32 up-regulated and 39 down-regulated metabolites, were identified by screening criteria of fold change (FC) >1.5 and *P*-value < 0.05. Data enrichment and metabolic pathway analysis were performed using Metabolanalyst 5.0. Ammonia recycling, glutathione metabolism, glutamate metabolism, and Beta A metabolism were enriched for differentially expressed metabolites of VCI (Figures 1A, B). As part of the metabolic signaling pathway enriched for aminoacyl-tRNA, glycine, serine, and threonine biosynthesis, valine, leucine, and isoleucine biosynthesis, glyoxylate and dicarboxylate metabolism, phenylalanine metabolism, phenylalanine, tyrosine, and tryptophan biosynthesis, etc (Figures 1A, B). Among the metabolic functions enriched for differentially expressed metabolites of HCC are the Warburg effect, gluconeogenesis, purine metabolism, phosphatidylethanolamine biosynthesis, phosphatidylcholine biosynthesis, and arginine and proline metabolism (Figures 1C, D). The main enriched metabolic signaling pathways include glycerophospholipid metabolism, citrate cycle (TCA cycle), pyruvate metabolism, purine metabolism, glycolysis/gluconeogenesis, and the alanine, aspartate, and glutamate metabolism (Figures 1C, D). Based on the above, it is evident that metabolism-related genes may play a role in HCC and VCI.

### HCC and VCI transcriptome DEGs

**Figure 2:**
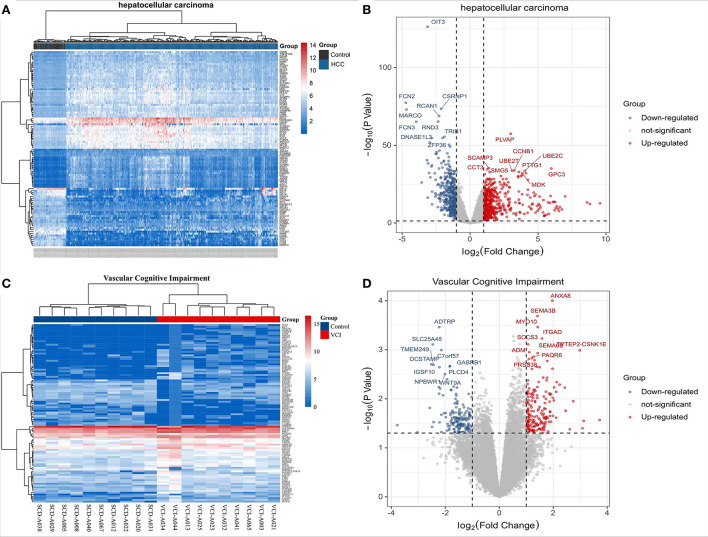
Transcriptomic analysis of HCC and VCI. **(A)** Heat map of DEGs clustering for HCC transcriptomics; **(B)** Transcriptomics of DEGs volcanoes for HCC; **(C)** Heat map of DEGs clustering for VCI transcriptomics; **(D)** Volcano map for VCI transcriptomics based on DEGs.

The TCGA website (https://portal.gdc.cancer.gov/) was used to download clinical and transcriptomic expression data related to HCC, and a total of 424 transcriptomic and 377 clinical datasets were obtained according to the predefined screening criteria. Differential expression data were screened based on adjusted *P*-values < 0.05 and |log_2_FC| ≥ 1.0. A total of 882 differentially expressed mRNAs were screened in the dataset, including 487 up-regulated mRNAs and 395 down-regulated mRNAs; DEGs were screened for differential analysis based on *P*-value. The top 100 most significant differentially expressed mRNAs were screened based on *P*-values and plotted as heat maps (Figure 2A). Following differential analysis, *P*-values were –log10 transformed, grouped according to log2 FC (groups of up-regulated DEGs, down-regulated DEGs, and non-statistically significant DEGs), and imported into R for plotting volcanoes (Figure 2B). The GSE201482 dataset contained 343 differentially expressed mRNAs, including 179 up-regulated and 164 down-regulated mRNAs. The top 100 differentially expressed coding RNAs with the greatest significance were screened, and the heat map was created based on the *P*-value (Figure 2C). Further, –log10 (*P*-value) was transformed from differential analysis microarray data, –log10 (*P*-value) was grouped by log2 FC (groups of up-regulated DEGs, down-regulated DEGs, and non-statistically significant DEGs), and the processed data was imported into R to plot volcanoes (Figure 2D).

### Multi-omics analysis of HCC and VCI

**Figure 3:**
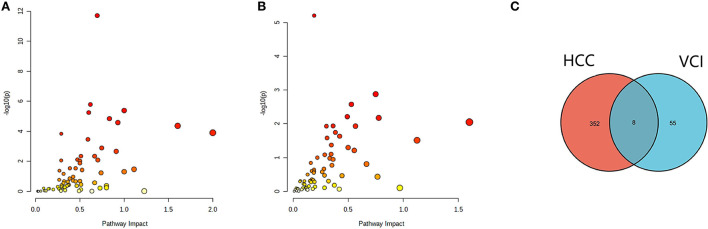
Analysis of multi-omics data; **(A)** Bubble map of enrichment distribution of HCC metabolism-related genes; **(B)** Bubble map of enrichment distribution of VCI metabolism-related genes; **(C)** Venn diagram showing the intersection of HCC and VCI differential genes.

**Figure 4:**
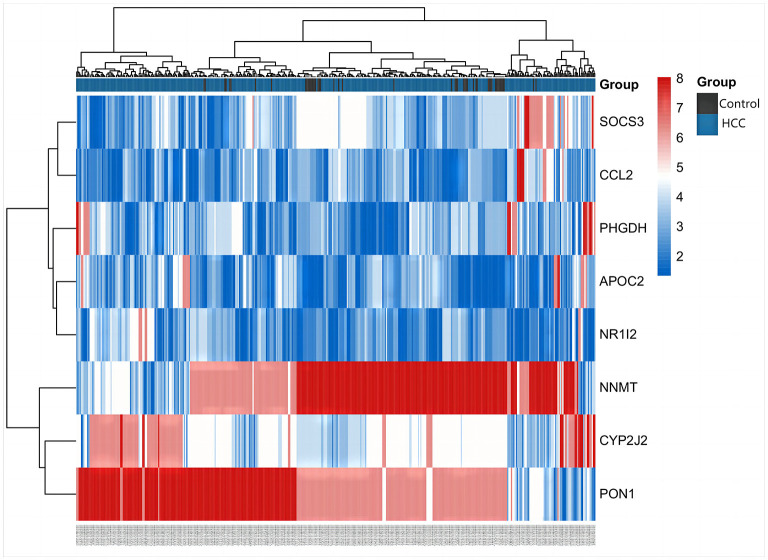
Clustering of metabolism-related genes in HCC.

The differentially expressed metabolites and DEGs of HCC and VCI obtained in the previous stage were imported into Metaboanalyst 5.0 for multi-omics analysis, and 360 metabolic DEGs associated with HCC and 63 metabolic DEGs associated with VCI were obtained (Figures 3A, B). To plot the Venn diagram, the above metabolomics genes associated with HCC and VCI were imported into Venny 2.1 software (http://bioinfogp.cnb.csic.es/tools/venny/index.html). Accordingly, 8 mRNAs were selected, including 1 up-regulated and 7 down-regulated mRNAs, namely: NNMT, PHGDH, NR1I2, CYP2J2, PON1, APOC2, CCL2, and SOCS3 (Figure 3C). Figure 4 shows a heat map of those differentially metabolized DEGs related to HCC and VCI.

### Prognostic models based on metabolic genes related to HCC and VCI

**Figure 5:**
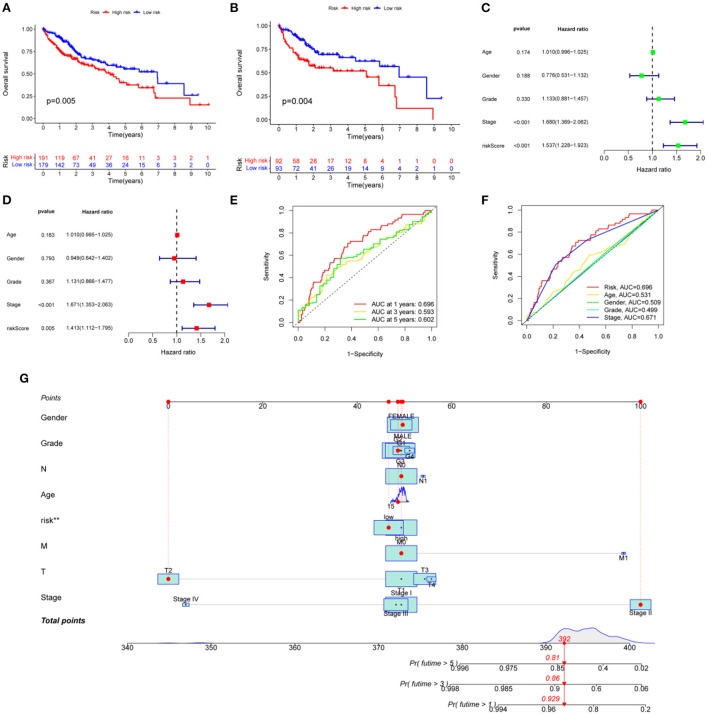
Developing a prognosis model for HCC. **(A)** Prognostic curves for the overall sample group; **(B)** The survival prognosis curve for the training group; **(C)** Analysis of independent prognostic factors based on single-factor regression; **(D)** Analysis of independent prognostic factors based on multi-factor regression; **(E)** HCC 5-year survival ROC curves; **(F)** HCC risk factor ROC curves; **(G)** Nomograph of metabolomic prognostic models for HCC.

The 377 clinical cases were randomly divided into a test group and a training group, for clinical and statistical analysis. No significant differences were found between the two groups in terms of age, gender, and tumor stage (*P*-value > 0.05), and the data from the two groups were comparable, as shown in Supplementary Table 1. Based on a multifactorial regression model, we composed these six genes (NNMT, PHGDH, NR1I2, CYP2J2, PON1, APOC2, CCL2, and SOCS3) into a risk factor model called riskScore. In the COX survival prognostic model, survival time decreased with increasing riskScore in the test group and the training group (*P*-value < 0.05) (Figures 5A, B). Independent prognostic analyses by univariate and multifactorial regression showed that the tumor stage and riskScore were significantly associated with prognosis in HCC patients (Figures 5C, D). For prognosis prediction of HCC patients at 1, 3, and 5 years, the area under the curve (AUC) of the subject working characteristic (ROC) curve exceeded 0.59 (Figure 5E). Among the ROC curves for all HCC risk factors, the AUC of riskScore for metabolic genes related to HCC and VCI was the largest and >0.6 (Figure 5F), indicating a good sensitivity of the established survival prognostic model. Based on HCC and VCI-related metabolic genes, a nomogram prognostic model was constructed, and the test results showed survival rates of 0.929, 0.86, and 0.81 after 1 year (*P*-value < 0.05) (Figure 5G).

### Principal component analysis, GO, and KEGG enrichment analysis of metabolism-related genes of HCC

**Figure 6:**
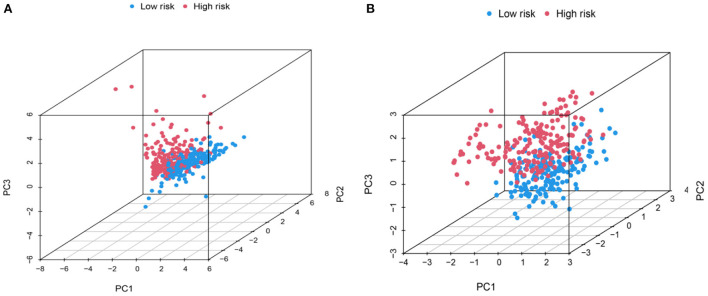
PCA distribution map. **(A)** Metabolic-related genes PCA distribution map; **(B)** Genes related to HCC PCA distribution map.

**Figure 7:**
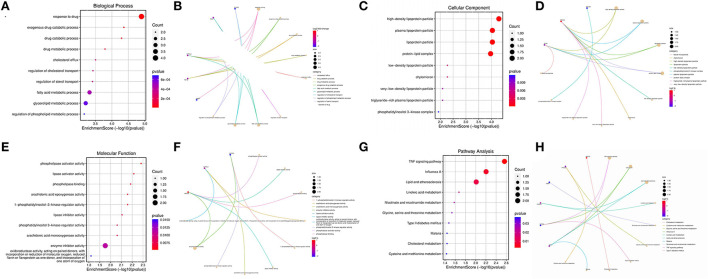
Enrichment analysis; **(A)** BP enrichment bubble plot; **(B)** BP enrichment plot; **(C)** CC enrichment bubble plot; **(D)** CC enrichment plot; **(E)** MF enrichment bubble plot; **(F)** MF enrichment plot; **(G)** KEGG enrichment bubble plot; **(H)** KEGG enrichment plot.

Principal component analysis of HCC metabolism-related genes and HCC-associated genes was performed using the scatterplot3d package (Figures 6A, B). GO and KEGG pathway enrichment analysis of those eight differentially metabolized DEGs related to HCC and VCI were done using Bioconductor package and clusterprofiler package in R language. The GO analysis of those eight differentially metabolized DEGs showed that their biological processes were mainly enriched in response to a drug or an exogenous drug catabolic process (Figures 7A–F). The cellular components that were mainly enriched included high-density lipoprotein particles and plasma lipoprotein particles. The molecular functions including phospholipase activator activity, lipase activator activity, and phospholipase binding were also enriched. The enriched KEGG pathways included TNF signaling pathway, Influenza A lipid and atherosclerosis pathway, linoleic acid metabolism, nicotinate and nicotinamide metabolism, glycine, serine, and threonine metabolism, and so on (Figures 7G, H). This indicates that the pathways and functions of these DEGs enrichment may be connected to the immune microenvironment and metabolism.

### Immuno-infiltration and immunofunctional analysis of metabolized DEGs related to HCC and VCI

**Figure 8:**
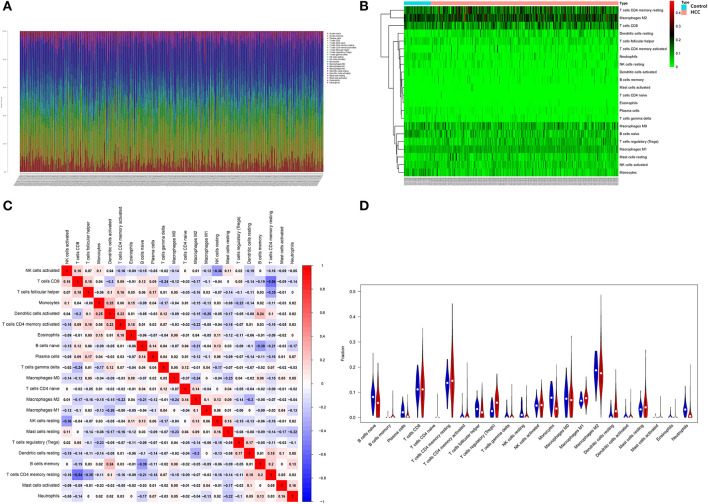
Infiltration analysis of immune cells. **(A)** Distribution of immune cells in HCC; **(B)** HCC immune cell distribution heat map; **(C)** HCC immune cell interaction heat map; **(D)** Image showing the relative amount of immune cells in HCC.

The obtained HCC expression matrix data were background corrected and normalized and expression values for the microarray data were calculated using the limma R package in R. The immune cell composition of each sample was further analyzed using the CIBERSORT package and histograms were plotted (Figure 8A). The immune cell distribution heat map was plotted using the pheatmap package (Figure 8B). The interaction of immune cell populations in HCC was then analyzed using the corrplot package and co-expression maps of immune cell infiltration in HCC were plotted (Figure 8C). Finally, the expression matrix data were analyzed using the Vioplot package to investigate the expression of each immune cell in HCC tissues as well as paracancerous tissues, and further plotted (Figure 8D).

**Figure 9:**
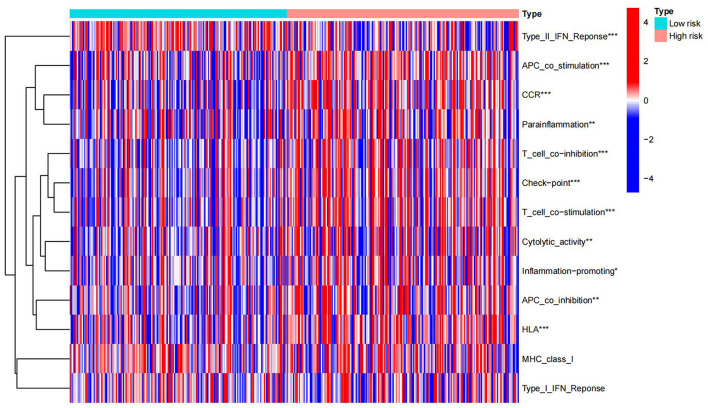
Immune enrichment analysis heat map.

Immunological functions of HCC metabolism-related genes were analyzed using limma, Gene Set Variation Analysis (GSVA), gseabase, pheatmap, and reshape2 packages. The immune functions were mainly focused on antigen-presenting cells (APC) co-inhibition, APC co-stimulation, cytokine-cytokine receptor interaction (CCR), checkpoint, cytolytic activity, human leukocyte antigens (HLA), inflammation-promoting, MHC_class_I, and parainflammation (Figure 9). This study reveals and visualizes immune infiltration and immune function associated with metabolic DEGs of HCC and VCI.

### TMB analysis of metabolized DEGs related to HCC and VCI

**Figure 10:**
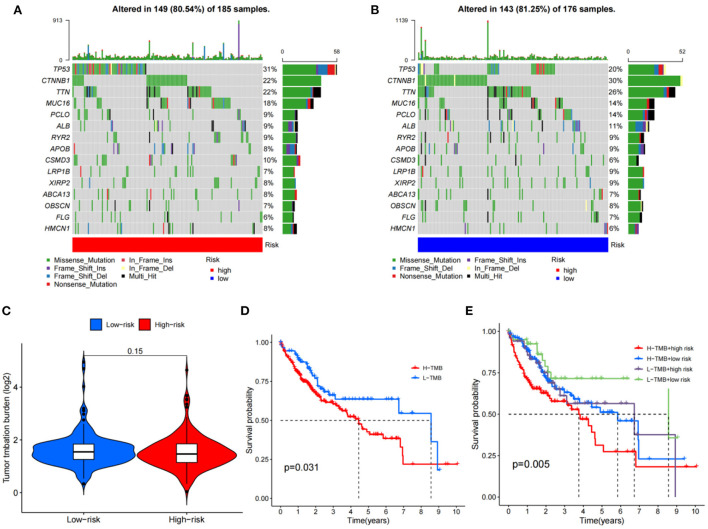
TMB analysis; **(A)** The waterfall plot for the high risk group; **(B)** The waterfall plot for the low risk group; **(C)** The differential analysis of tumor mutation burden; **(D)** Survival analysis of tumor mutation burden; **(E)** Survival analysis of TMV+ risk factors.

The HCC expression data and TMB files were imported into R, and the correlation between DEGs related to HCC and tumor mutation load was calculated by using the function, and the waterfall plots of high and low-risk groups were drawn according to the correlation results (Figures 10A, B). The differential analysis results suggested that the tumor mutation load in the low-risk group was significantly higher than that in the high-risk group (Figure 10C). Survival analysis of the tumor mutation load of HCC metabolism-related genes showed that the survival period of the low tumor mutation load group was significantly longer than that of the high tumor mutation load group (*P*-value < 0.05) (Figure 10D). In addition, combining tumor mutation load characteristics and metabolism-related genetic factors, the low-risk group with low tumor mutation load had the highest probability of survival while the high-risk group with low tumor mutation load had the lowest probability of survival (Figure 10E).

### Relative expression of PHGDH, NR1I2, and APOC2

**Figure 11:**
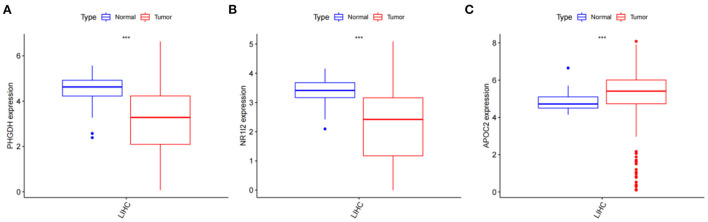
Analysis of the relative expression of target genes; **(A)** Relative expression of PHGDH (*p* < 0.001); **(B)** Relative expression of NR1I2 (*p* < 0.001); **(C)** Relative expression of APOC2 (*p* < 0.001).

After obtaining the core genes PHGDH, NR1I2, and APOC2 from the previous differential expression analysis and survival prognosis analysis, we further investigated their relative expression in HCC. The transcriptome data of HCC were downloaded from the TCGA and the ggpubr package was used to analyze the relative expression of the identified core genes and to draw box expression maps (Figures 11A–C). The results revealed that PHGDH and NR1I2 genes exhibited low expression in HCC tumor tissues *(P*-value < 0.001) while APOC2 genes were highly expressed in HCC tumor tissues (*P*-value < 0.01).

### Single-gene GSEA enrichment analysis

**Figure 12:**
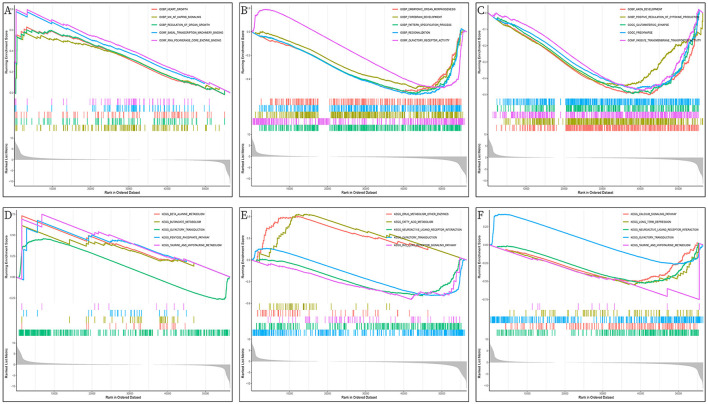
GSEA enrichment analysis of DEGs (*PHGDH, NR1I2*, and *APOC2*); **(A)** GO enrichment analysis of *PHGDH* in HCC; **(B)** GO enrichment analysis of *NR1I2* in HCC; **(C)** GO enrichment analysis of *APOC2* in HCC; **(D)** KEGG enrichment analysis of *PHGDH* in HCC; **(E)** KEGG enrichment analysis of *NR1I2* in HCC; **(F)** KEGG enrichment analysis of *APOC2* in HCC.

The GO/KEGG annotation file downloaded from the GSEA website and the HCC tumor data file were read into R. Analytical operations were performed, and it was found that: the GO of gene *PHGDH* at HCC was enriched in CHROMATIN REMODELING, DNA PACKAGING, and PROTEIN DNA COMPLEX SUBUNIT ORGANIZATION functions (Figure 12A). The GO function of gene *NR1I2* was enriched in ACTIVATION OF IMMUNE RESPONSE, ADAPTIVE IMMUNE RESPONSE BASED ON SOMATIC RECOMBINATION OF IMMUNE RECEPTORS BUILT FROM IMMUNOGLOBULIN SUPERFAMILY DOMAINS, and ALPHA BETA T CELL ACTIVATION (Figure 12B). The gene *APOC2* was functionally enriched in CHROMATIN ASSEMBLY OR DISASSEMBLY, EPIDERMAL CELL DIFFERENTIATION, and INFLAMMATORY RESPONSE TO ANTIGENIC STIMULUS (Figure 12C); The main PHGDH gene-enriched KEGG pathways are OLFACTORY TRANSDUCTION, CIRCADIAN RHYTHM MAMMAL, GRAFT VS. HOST DISEASE signaling pathways (Figure 12D); The *NR1I2* gene enriched KEGG pathways are mainly ANTIGEN PROCESSING AND PRESENTATION, CYTOKINE RECEPTOR INTERACTION, and CYTOSOLIC DNA SENSING signaling pathways (Figure 12E). The main KEGG pathways enriched by the *APOC2* gene are OLFACTORY TRANSDUCTION, CYTOSOLIC DNA SENSING PATHWAY, and REGULATION OF AUTOPHAGY signaling pathway (Figure 12F).

### Potential drug screening

**Figure 13:**
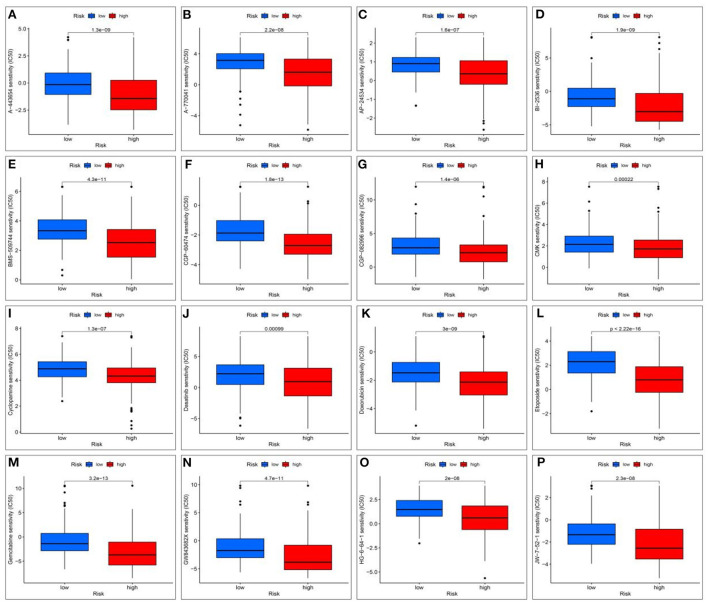
Screening for potential drugs; **(A)** The therapeutic effect of the drug A-443654; **(B)** The therapeutic effect of the drug A-770041; **(C)** The therapeutic effect of the drug AP-24534; **(D)** The therapeutic effect of the drug BI-2536; **(E)** The therapeutic effect of the drug BMS-509744; **(F)** The therapeutic effect of the drug CGP-60474; **(G)** The therapeutic effect of the drug CGP-082996; **(H)** The therapeutic effect of the drug RSK2 kinase inhibitor (CMK); **(I)** Cyclopamine drug's therapeutic effects; **(J)** The therapeutic effect of the drug Dasatinib; **(K)** The therapeutic effect of the drug Doxorubicin; **(L)** The therapeutic effect of the drug Etoposide; **(M)** The therapeutic effect of the drug Gemcitabine; **(N)** The therapeutic effect of the drug GW843682X; **(O)** The therapeutic effect of the drug HG-6-64-1; **(P)** The therapeutic effect of the drug HG-6-64-1.

To evaluate therapeutically effective drugs against DEGs related to HCC and VCI, we used the limma, ggpubr, prrophetic, and ggplot2 packages. Drugs with potential clinical efficacy against HCC metabolism-related genes include A-443654, A-770041, AP-24534, BI-2536, BMS-509744, CGP-60474, CGP-082996, CMK, cyclopamine, dasatinib, doxorubicin, etoposide, gemcitabine, GW843682X, HG-6-64-1, and JW-7-52-1 (Figures 13A–P).

## Discussions

VCI is an acquired mental impairment syndrome, which is characterized by cognitive impairment, memory difficulties, and neurodegenerative lesions (44). Previous studies have pointed out that HCC may be associated with cognitive disorders of the brain such as hepatic encephalopathy, but the specific mechanisms by which HCC induces VCI are unclear (45). Metabolomic studies have shown that the pathogenesis of VCI may involve various mechanisms such as impaired myelin synthesis caused by glucolipid metabolism disorders and related metabolites leading to blood-brain barrier disruption and vascular endothelial damage (46–48). HCC may affect metabolic disorders promoting the development of VCI. According to the current studies, the possible mechanisms of cognitive impairment due to HCC include impaired blood ammonia and bile acid metabolism, oxidative stress injury and inflammatory response, impaired blood-brain barrier, neurotransmission disorders, neurotoxic accumulation, and disturbance of cerebral energy metabolism (49–51). Therefore, in this study, we propose that HCC may influence the body's metabolism to promote the occurrence of VCI. In this study, we obtained the 8 significant HCC-VCI DEGs by metabolomic analysis, which are NNMT, PHGDH, NR1I2, CYP2J2, PON1, APOC2, CCL2, and SOCS3. Previous literature indicated that the above eight genes are mostly involved in the development of VCI.

NNMT encodes proteases involved in the metabolism of various substances and drugs *in vivo* and is highly expressed in a variety of tumors, correlating with tumor infiltration, distant metastasis, and malignancy (52, 53). NNMT encodes neuronal proteins whose increased expression is associated with stress responses in the brain microenvironment and can be involved in promoting cognitive impairment (54–56). PHGDH encodes a phosphoglycerate dehydrogenase involved in the synthesis of L-serine, a regulator of synaptic plasticity and an essential product for T-cell expansion (57, 58). PHGDH mutants can result in neurological symptoms such as impaired motor function, and its overexpression can promote proliferation and invasion of cancer cells such as HCC (59–61). Further, PHGDH has been found to contribute to the development of cognitive impairment (62). NR1I2 is a member of the nuclear receptor superfamily and is involved in encoding a transcriptional regulator that regulates cytochrome P450 (CYP) enzymes. Previous studies suggest that NR1I2 may regulate bile acid metabolism involved in promoting the progression of cognitive impairment (63). CYP2J2 is a cyclooxygenase that is involved in regulating the body's inflammatory response, cell proliferation, and other physiological functions, and plays an important role in the homeostasis (64). CYP2J2 variants are involved in promoting cerebrovascular disease and their polymorphisms are associated with susceptibility to cognitive impairment (65, 66). CYP2J2 increases the production of eets and enhances HIF-1 alpha stability and promotes the development of HCC (67). PON1 encodes calcium-dependent high-density lipoprotein-related lipase, which can reduce reactive oxygen species, reduce LDL oxidative stress, enhance HDL antioxidant capacity, and participate in anti-inflammatory and antioxidant activities in neurodegenerative diseases, neuroinflammation, and other neurological diseases (68, 69). Studies have pointed out that reduced PON1 activity may affect lipid metabolism, promote vascular endothelial damage, and contribute to the development of cognitive impairment (69–72). PON1 levels were negatively correlated with HCC vascular invasion, probably due to its anti-inflammatory activity and its role in the maintenance of normal vascular endothelial function (73). APOC2 encodes a lipid-binding protein belonging to the apolipoprotein family, which is involved in the composition of very low-density lipoproteins and in promoting the hydrolysis of triglycerides (74, 75). High serum levels of APOC2 are associated with cognitive impairment, and the exact underlying mechanism of action is unclear (76, 77). CCL2 or MCP-1 is a member of the CC chemokine family, and abnormally expressed CCL2 is closely associated with CNS diseases, neoplastic diseases, and inflammatory diseases. The CCL2/CCR2 axis activates the Hedgehog pathway involved in the induction of HCC invasion and epithelial-mesenchymal transition (78). Further, CCL2 may be involved in promoting the progression of cognitive impairment through enhancing excitotoxicity, oxidative stress-induced inflammatory damage, and apoptosis in neuronal cells, affecting glutamate metabolism and inducing microglia activation in the local microenvironment (79–82). SOCS3 is an important negative feedback regulatory protein in the JAK/STAT signaling pathway, a key physiological regulator in natural and acquired immunity, T-lymphocyte differentiation, and immune regulation, and plays a negative feedback regulatory role in immune/inflammatory diseases (83–85). The SOCS3 signaling pathway is also involved in the development of HCC (86, 87). Thus, the above genes may be associated with the occurrence of HCC-induced VCI.

The developed HCC metabolomics prognostic model based on HCC-VCI DEGs has a good prognostic effect. Principal component analysis, functional enrichment analysis, immune function analysis, and TMB analysis suggested that HCC-VCI DEGs may affect the immune microenvironment and thus result in the occurrence and development of VCI in HCC. Therefore, the immune-related effects of the above genes were probed, and it was observed that several of these genes were associated with immunity. PHGDH can promote liver ceramide synthesis to maintain lipid homeostasis, and participate in the maintenance of mitochondrial REDOX homeostasis and cellular homeostasis in the immune microenvironment (88, 89). NR1I2 is involved in the regulation of T-cell differentiation and plays an important role in the regulation of immune homeostasis (90, 91). APOC2 may play a protective role in the immune microenvironment of atherosclerotic disease by inhibiting foam cell formation, in addition to participating in lipid metabolism (74, 92, 93). CCL2 plays an important role in immune regulation by recruiting chemotactic monocytes/macrophages to the site of inflammation, mediating the inflammatory response to limit pathogen invasion, and participating in the repair of damaged tissues (94, 95).

In summary, our study revealed that the HCC-associated metabolic DEGs such as NNMT and PHGDH may influence the occurrence and progression of VCI in HCC patients by affecting the immune microenvironment. Further by establishing a prognostic model and screening potential targeted drugs, we found that the above genes had a good prognostic effect on HCC. Finally, drugs that may target HCC-VCI DEGs were screened, namely A-443654, A-770041, AP-24534, BI-2536, BMS-509744, CGP-60474, and CGP-082996, and are expected to have good potential clinical efficacy in patients with HCC-induced VCI. Therefore, this study provides theoretical support and potential therapeutic strategies for the pathogenesis and clinical treatment of VCI in HCC patients. However, the current study also has some limitations. On the one hand, a larger sample of data is needed to verify the conclusions while on the other, relevant biological experiments are needed to clarify the specific regulatory mechanisms.

## Conclusion

HCC metabolization-related DEGs such as NNMT and PHGDH may lead to the occurrence of VCI in HCC patients by affecting the immune microenvironment. Further by establishing a prognostic model and screening the potential targeted drugs, we found that the above genes had a good prognostic effect on HCC. A-443654, A-770041, AP-24534, BI-2536, BMS-509744, CGP-60474, and CGP-082996 could be potential candidates with good clinical efficacy for treating VCI in HCC patients.

## Data availability statement

The original contributions presented in the study are included in the article/Supplementary material, further inquiries can be directed to the corresponding author.

## Author contributions

DZ, YZ, LL, XH, and SF contributed to conception and design of the study and organized the database. DZ and SF performed the statistical analysis and wrote sections of the manuscript. DZ wrote the first draft of the manuscript. All authors contributed to manuscript revision, read, and approved the submitted version.
