# Supplementary material for: Metabolomic analysis of vascular cognitive impairment due to hepatocellular carcinoma
Source: Front Neurol. 2023 Mar 16;13:1109019. doi: 10.3389/fneur.2022.1109019 (PMC10062391; doi:10.3389/fneur.2022.1109019)
Supplement: Supplementary file 1 [file Table_1.DOCX]

**Supplementary Table 1. Clinical information about patients in the test and training groups**

| **Covariates** | **Type** | **Total** | **Test group** | **Train group** | ***P-value*** |
| --- | --- | --- | --- | --- | --- |
| Age | <=65 | 232(62.7%) | 116(62.7%) | 116(62.7%) | 1 |
|  | >65 | 138(37.3%) | 69(37.3%) | 69(37.3%) |  |
| Gender | FEMALE | 121(32.7%) | 65(35.14%) | 56(30.27%) | 0.375 |
|  | MALE | 249(67.3%) | 120(64.86%) | 129(69.73%) |  |
| Grade | G1 | 55(14.86%) | 26(14.05%) | 29(15.68%) | 0.819 |
|  | G2 | 177(47.84%) | 87(47.03%) | 90(48.65%) |  |
|  | G3 | 121(32.7%) | 64(34.59%) | 57(30.81%) |  |
|  | G4 | 12(3.24%) | 7(3.78%) | 5(2.7%) |  |
|  | unknow | 5(1.35%) | 1(0.54%) | 4(2.16%) |  |
| Stage | Stage I | 171(46.22%) | 83(44.86%) | 88(47.57%) | 0.609 |
|  | Stage II | 85(22.97%) | 47(25.41%) | 38(20.54%) |  |
|  | Stage III | 85(22.97%) | 39(21.08%) | 46(24.86%) |  |
|  | Stage IV | 5(1.35%) | 2(1.08%) | 3(1.62%) |  |
|  | unknow | 24(6.49%) | 14(7.57%) | 10(5.41%) |  |
| T | T1 | 181(48.92%) | 89(48.11%) | 92(49.73%) | 0.599 |
|  | T2 | 93(25.14%) | 52(28.11%) | 41(22.16%) |  |
|  | T3 | 80(21.62%) | 37(20%) | 43(23.24%) |  |
|  | T4 | 13(3.51%) | 6(3.24%) | 7(3.78%) |  |
|  | unknow | 3(0.81%) | 1(0.54%) | 2(1.08%) |  |
| M | M0 | 266(71.89%) | 135(72.97%) | 131(70.81%) | 1 |
|  | M1 | 4(1.08%) | 2(1.08%) | 2(1.08%) |  |
|  | unknow | 100(27.03%) | 48(25.95%) | 52(28.11%) |  |
| N | N0 | 252(68.11%) | 119(64.32%) | 133(71.89%) | 1 |
|  | N1 | 4(1.08%) | 2(1.08%) | 2(1.08%) |  |
|  | unknow | 114(30.81%) | 64(34.59%) | 50(27.03%) |  |
